# Development and validation of a prognostic model for the early identification of COVID-19 patients at risk of developing common long COVID symptoms

**DOI:** 10.1186/s41512-022-00135-9

**Published:** 2022-11-17

**Authors:** Manja Deforth, Caroline E. Gebhard, Susan Bengs, Philipp K. Buehler, Reto A. Schuepbach, Annelies S. Zinkernagel, Silvio D. Brugger, Claudio T. Acevedo, Dimitri Patriki, Benedikt Wiggli, Raphael Twerenbold, Gabriela M. Kuster, Hans Pargger, Joerg C. Schefold, Thibaud Spinetti, Pedro D. Wendel-Garcia, Daniel A. Hofmaenner, Bianca Gysi, Martin Siegemund, Georg Heinze, Vera Regitz-Zagrosek, Catherine Gebhard, Ulrike Held

**Affiliations:** 1 Department of Biostatistics at Epidemiology, Biostatistics and Prevention Institute, University of Zurich, Zurich, Switzerland; 2 Intensive Care Unit, Department of Acute Medicine, University Hospital Basel, Basel, Switzerland; 3 University of Basel, Basel, Switzerland; 4 Department of Nuclear Medicine, University Hospital Zurich, Zurich, Switzerland; 5 Center for Molecular Cardiology, University of Zurich, Schlieren, Switzerland; 6 Institute of Intensive Care Medicine, University Hospital Zurich, Zurich, Switzerland; 7 University of Zurich, Zurich, Switzerland; 8 Department of Infectious Diseases and Hospital Epidemiology, University Hospital Zurich, Zurich, Switzerland; 9 Department of Internal Medicine, Cantonal Hospital Baden, Baden, Switzerland; 10 Department of Infectiology and Infection Control, Cantonal Hospital Baden, Baden, Switzerland; 11 Department of Cardiology, University Hospital Basel, Basel, Switzerland; 12 University Center of Cardiovascular Science & Department of Cardiology, University Heart and Vascular Center Hamburg, University Medical Center Hamburg-Eppendorf, Hamburg, Germany; 13 German Center for Cardiovascular Research (DZHK) Partner Site Hamburg-Kiel-Lübeck, Berlin, Germany; 14 Department of Intensive Care Medicine, University Hospital Bern, Bern, Switzerland; 15 Department Clinical Research, University of Basel, Basel, Switzerland; 16 Center for Medical Statistics, Informatics and Intelligent Systems, Section for Clinical Biometrics, Medical University of Vienna, Vienna, Austria; 17 Charité, University Medicine Berlin, Berlin, Germany; 18 Department of Cardiology, University Hospital Zurich, Zurich, Switzerland

**Keywords:** Clinical prediction model, Long COVID, Prognostic factors, Stratified medicine

## Abstract

**Background:**

The coronavirus disease 2019 (COVID-19) pandemic demands reliable prognostic models for estimating the risk of long COVID. We developed and validated a prediction model to estimate the probability of known common long COVID symptoms at least 60 days after acute COVID-19.

**Methods:**

The prognostic model was built based on data from a multicentre prospective Swiss cohort study. Included were adult patients diagnosed with COVID-19 between February and December 2020 and treated as outpatients, at ward or intensive/intermediate care unit. Perceived long-term health impairments, including *r*educed exercise tolerance/reduced r*e*silience, *s*hortness of breath and/or *t*iredness (REST), were assessed after a follow-up time between 60 and 425 days. The data set was split into a derivation and a geographical validation cohort. Predictors were selected out of twelve candidate predictors based on three methods, namely the augmented backward elimination (ABE) method, the adaptive best-subset selection (ABESS) method and model-based recursive partitioning (MBRP) approach. Model performance was assessed with the scaled Brier score, concordance *c* statistic and calibration plot. The final prognostic model was determined based on best model performance.

**Results:**

In total, 2799 patients were included in the analysis, of which 1588 patients were in the derivation cohort and 1211 patients in the validation cohort. The REST prevalence was similar between the cohorts with 21.6% (*n* = 343) in the derivation cohort and 22.1% (*n* = 268) in the validation cohort. The same predictors were selected with the ABE and ABESS approach. The final prognostic model was based on the ABE and ABESS selected predictors. The corresponding scaled Brier score in the validation cohort was 18.74%, model discrimination was 0.78 (95% CI: 0.75 to 0.81), calibration slope was 0.92 (95% CI: 0.78 to 1.06) and calibration intercept was −0.06 (95% CI: −0.22 to 0.09).

**Conclusion:**

The proposed model was validated to identify COVID-19-infected patients at high risk for REST symptoms. Before implementing the prognostic model in daily clinical practice, the conduct of an impact study is recommended.

**Supplementary information:**

The online version contains supplementary material available at 10.1186/s41512-022-00135-9.

## Background

The World Health Organization (WHO) reported over 571 million confirmed coronavirus disease 2019 (COVID-19) infections globally through to the end of July 2022 [1]. The acute COVID-19 infection numbers and subsequent treatments cause a high burden for the health care system, an economic burden and a potential burden to the patient. A COVID-19 infection may lead to long-term health effects [2, 3]. Nowadays different definitions of long-term conditions exist. The WHO defines post COVID-19 with symptoms for at least 3 months after the acute COVID-19 infection [4]. The National Institute for Health and Care Excellence (NICE) of England, the Scottish Intercollegiate Guidelines Network and the Royal College of General Practitioners of the United Kingdom distinguish between post-COVID-19 syndrome and ongoing symptomatic COVID-19 [5]. Post-COVID-19 refers to symptoms persisting for longer than 12 weeks, whereas ongoing symptomatic COVID-19 is defined by symptoms lasting between 4 and 12 weeks. Ongoing symptoms and post-COVID-19 are often described as long COVID [5]. According to an umbrella review, the prevalence of long COVID in adults ranges from 2.3 to 53.0% [3].

A prognostic model would allow to identify the patients at risk of developing common long COVID symptoms. Prognostic models are part of the stratified or personalized medicine, which aims at tailoring therapies to patients by considering individual patients’ characteristics as for example genetics, psychosocial or biological factors [6, 7].

Due to the potential usefulness of prognostic models, it is not astonishing that the number of publications aiming at deriving prognostic models has increased in recent years [8]. However, several systematic reviews raise doubts about the reliability of published prognostic models. High risk of bias, lack of validation and insufficient reporting were identified across different diseases [9–14]. For implementing higher standards of prognostic models’ quality and facilitating reporting, the Transparent Reporting of a Multivariable Prediction Model for Individual Prognosis or Diagnosis (TRIPOD) statement and checklist was developed and published in 2015 [15, 16].

The COVID-19 pandemic demands for reliable prognostic models for estimating the risk of long COVID. The main objective of this study was to develop and validate a prognostic model for the probability estimation of common long COVID symptoms between 60 and 425 days after acute COVID-19 infection in adult patients of a multicentre prospective Swiss cohort study.

## Methods

This study was conducted and reported following the recommendations of the TRIPOD statement [15, 16]. The local ethics committee approved this study (BASEC #2020-01311).

### Study design and study population

In total, 2799 patients of a multicentre prospective Swiss cohort study were included in the study. The prognostic model was developed with data from 1588 patients (1395 outpatients and 193 hospitalized patients) and validated with 1211 patients (941 outpatients and 270 hospitalized patients). In-hospital patients from either ward and/or intensive/intermediate care unit (ICU/IMC) were eligible. The patients were at least 18 years old, diagnosed with COVID-19 (positive SARS-CoV-2 PCR test) at one of four Swiss hospitals, namely the University Hospital Basel, University Hospital Bern, University Hospital Zurich and Cantonal Hospital Baden, between February and December 2020. After a follow-up time between 60 and 425 days, the patients answered a questionnaire. The questionnaire contained questions regarding the patient’s characteristics (for example sex, age, body mass index), symptoms and severity of the acute COVID-19 infection, comorbidities, cardiovascular risk factors and questions regarding the personal situation during the COVID-19 pandemic. The questionnaire was available in German, French, Italian and English.

### Cohort data for derivation and validation

According to the sample size calculation, the data set is large enough to split the data into a derivation and validation cohort. The data for model development and validation was separated based on geographical reasoning. A geographic validation instead of a standard cross-validation was chosen because a geographic validation is more meaningful [17]. The derivation cohort, used for the model development, was formed by patients recruited at the University Hospital Basel. The validation cohort contained the patients from University Hospital Zurich, University Hospital Bern and Cantonal Hospital Baden.

### Outcome definition and follow-up

More than 50 symptoms have been described to be potentially related to long COVID [3]. We defined a composite outcome, addressing three long COVID symptoms, namely *r*educed exercise tolerance/reduced r*e*silience, *s*hortness of breath and *t*iredness (REST). The focus was placed on the REST symptoms, because the symptoms are potentially treatable/modifiable, are common [2, 3] and represent a significant burden on the patient. The binary REST outcome, encoded with 0 (no REST symptoms) and 1 (at least one of three REST symptoms), was assessed at follow-up. The follow-up was defined to be at least 60 days after acute COVID-19 infection.

### Candidate predictors

Twelve candidate predictors including patient’s demographic information, their number of comorbidities, the presence of cardiovascular risk factors, the number of symptoms during acute COVID-19, the severity of acute COVID-19 and gender-related characteristics were considered for inclusion in the prognostic model. A detailed description of the candidate predictors and their coding is given below:

- Patient demographics consisting of sex (1 female, 0 male), age at presentation and body mass index
- Number of comorbidities, i.e. the sum of the following pre-existing diseases prior to COVID-19 infection. Each pre-existing disease was coded with 0 (absent disease) or 1 (present disease, e.g., maximum 1 point for heart disease):
  - Heart disease: Heart failure, narrowing of the coronary arteries and/or heart attack, congenital heart disease, heart muscle inflammation (myocarditis)
  - Vascular disease: deep vein thrombosis in the leg, pulmonary embolism, stroke, blood clotting disorder, blood disease
  - Kidney disease
  - Diseases of the immune system including autoimmune disease
  - Lung disease: asthma, chronic obstructive pulmonary disease, pulmonary hypertension
  - Nervous system disease
  - Liver disease
  - Infectious disease including human immunodeficiency virus and hepatitis
  - Malignant cancer currently or within the last five years
  - Psychiatric disease
  - Rheumatic disease
- Cardiovascular risk factors including diabetes, high blood pressure, increased cholesterol and/or family history, coded with 1 (at least one condition present) or 0 (no condition present)
- Candidate predictors related to acute COVID-19 infection:
  - Absolute number of symptoms including fever, shortness of breath, coughing, loss of smell, loss of sense of taste, gastro-intestinal problems, physical weakness, tiredness, headache and other symptoms. The answer “I don’t remember” was counted as one symptom.
  - Severity categorized in outpatients, hospitalized patients to normal ward and/or intensive or intermediate care unit (ICU/IMC), coded with 1 outpatients, 2 normal ward and 3 ICU/IMC.
- Gender-related candidate predictors:
  - Responsibility for childcare/family member on a numeric rating scale from 1 (no responsibility or not applicable/low risk) to 6 (full responsibility/high risk).
  - Being the main responsible person for the household as factor candidate predictor with 1 (no/low risk), 2 (the partners contribute to roughly equal shares) and 3 (yes or I live alone/high risk).
  - Feeling of stress at home measured on a numeric rating scale ranging from 1 (no stress/low risk) to 10 (maximum stress/high risk).

The candidate predictors were chosen based on pre-existing medical knowledge and discussions with clinical experts. With the exception of the severity of acute COVID-19 infection and age at presentation, the candidate predictors were obtained by questionnaire at follow-up. The patients provided information on their health status, and no clinical measurements were collected. The severity of acute COVID-19 infection as well as the patient’s age at first presentation was ascertained based on the patient file.

### Sample size

The minimum sample size for developing and validating a prognostic model was calculated following the recommendations of Riley et al. [18, 19]. For the prediction of the REST outcome, with an observed prevalence of approximately 22% in the derivation cohort, an estimated variance explained by the prognostic model of 15% [18], an assumed shrinkage factor of 0.932 and twelve candidate predictors (10 variables), 1006 patients are required. This corresponds to approximately 222 events and an event per predictor parameter ratio equal to 18 in the derivation cohort. For validation, a sample size of 988 patients is required. A calibration slope of 1, a corresponding 95% confidence interval width of 0.35 and a calibration intercept of 0 were assumed. The parameters $I_\alpha$ = 0.140, $I_{\alpha ,\beta }$ = −0.154 and $I_\beta$ = 0.297 (for explanations reference is made to [19]) were calculated based on the linear predictors, based on the prediction model from the derivation cohort.

### Missing data

Very few missing values in the candidate predictors were observed (max 1.6% in the variable responsibility for childcare/family member). They were replaced with a non-parametric iterative imputation method using a random forest algorithm [20]. In brief, the missing values from all candidate predictors are replaced temporarily. The iterative imputation methods starts with the candidate predictor $x_s$ containing the smallest proportion of missing values. A random forest with the non-missing values of $x_s$ as dependent variable and all candidate predictors as independent variables is built. Based on the random forest, the missing values of $x_s$ are imputed. The iterative imputation continues with the next candidate predictor and stops when a stopping criterion is reached. The iterative imputation method was performed on the derivation, validation and total cohort with the R package MissForest [21]. Missingness at random was assumed.

### Statistical analysis methods

Candidate predictors were summarized with descriptive statistics, with median and interquartile range (IQR) for continuous candidate predictors and counts and percentage for categorical candidate predictors by cohort. The standardized mean difference (SMD) between the derivation and validation cohort was calculated. A SMD greater than 0.1 was considered as indicating imbalance [22].

The prognostic model was developed by (I) selecting relevant predictors by applying three methods and (II) estimating the parameters at the selected relevant predictors. Based on the validation cohort, the (III) three models were geographically validated and the (IV) final prognostic model was determined. A detailed description of the four steps is given in the following.

#### I) Selecting relevant predictors

A global logistic regression model was fitted with the REST outcome as dependent variable and all candidate predictors as independent variables. Interactions between age and number of comorbidities, age and body mass index, age and responsibility for childcare/family member, sex and presence of at least one cardiovascular risk factor, and body mass index and number of comorbidities were included in case of evidence for an interaction (*p*-value ≤ 0.05). Interactions were preselected based on clinical reasoning. The assumptions of the logistic regression model were checked with leverage plots and Tukey-Anscombe plots. Nonlinear relationships between individual continuous candidate predictors and the REST outcome were investigated visually by fitting a generalized additive model [23]. The generalized additive model was fitted with the REST outcome as dependent variable and all candidate predictors as independent variables, with smooth functions (penalized regression splines) for the continuous or ordinal candidate predictors age, body mass index, responsibility for childcare/family member and feeling of stress at home. Collinearity may reduce the accuracy of the estimated coefficients [17], and for this reason, linear relationships between continuous candidate predictors were assessed by calculating Pearson’s correlation *r* and the variance inflation factor (VIF) [24]. Values of |r| $\ge$ 0.7 and/or VIF $\ge$ 5 were considered as problematic [25]. A high VIF or *r* indicates that the calculated regression coefficients are unstable. Variable selection was performed based on three methods, including augmented backward elimination (ABE) [26], adaptive best-subset selection (ABESS) [27] and model-based recursive partitioning (MBRP) [28]. In the following, each method is described.

##### ABE

ABE combines the backward elimination method with the change-in-estimate criterion. A detailed description of the method is given by Dunkler et al. [26]. In brief, ABE starts with the global logistic model. More important predictors can be predefined as ‘passive’ candidate predictors, less important predictors as ‘active’ candidate predictors. ABE will only be performed on active candidate predictors or candidate predictors of unknown importance. Backward elimination can be based on the significance level $\alpha$, Akaike’s information criterion (AIC) or Bayesian information criterion (BIC). Candidate predictors not selected with backward elimination will be evaluated further with the change-in-estimate criterion. The change-in-estimate criterion evaluates the change in the coefficient of a passive candidate predictor by removing an active candidate predictor repeatedly. By applying backward elimination only, an important predictor might falsely be excluded. The additional change-in-estimate criterion in the ABE method minimizes this risk. Backward elimination was conducted based on AIC and with the threshold of the relative change-in-estimate criterion set equal to 0.05 [26] by using the R package abe. Candidate predictors were neither specified as ‘passive only’ nor ‘active only’ variables. In other words, all candidate predictors were classified as predictors of unknown importance.

##### ABESS

The algorithm selects a few relevant predictors out of a set of candidate predictors, so that the corresponding prognostic model has the highest prediction accuracy [27]. For this, the algorithm builds an initial first set $S_1$, consisting of candidate predictors that are most correlated with the outcome. The remaining candidate predictors form a second set $S_2$. Less important candidate predictors from $S_1$ are replaced iteratively with relevant candidate predictors from $S_2$ by the ABESS algorithm until the model error is minimized. The most suitable model sparsity level is determined by a data-driven procedure using the special information criterion developed for ABESS. The algorithm for the best-subset selection problem is implemented in R with the package abess and the function abess.

##### MBRP

The MBRP algorithm builds a decision tree [28]. In brief, the algorithm starts by first fitting a logistic regression model to the full derivation cohort. Model parameter instabilities are assessed. In case of instabilities in candidate predictor estimates, the derivation cohort will be split into two child nodes at the highest instability. The split point that locally optimizes the negative log-likelihood is determined and one logistic regression model per node (in other words per split) is fitted. The procedure is repeated in each child node until there is no further evidence for parameter instability. The MBRP algorithm can handle nonlinear relationships and interactions between candidate predictors. All predictors could potentially be used for partitioning. In R, the MBRP algorithm is implemented in the function glmtree of the package partykit.

#### II) Estimation of parameters

The logistic regression model $\text {M}_\text {ABE}$ and $\text {M}_\text {ABESS}$ was fitted containing all selected predictors found with the ABE and ABESS method, respectively, as independent variables, and the REST outcome as dependent variable. Non-linear and non-additive (interaction) effects were investigated [26]. The logistic regression model $\text {M}_\text {MBRP}$ was fitted containing the selected predictors found with the MBRP approach as independent variables and the REST outcome as dependent variable.

#### III) Model validation

The model performance of $\text {M}_\text {ABE}$, $\text {M}_\text {ABESS}$ and $\text {M}_\text {MBRP}$ was assessed by evaluating the overall performance, the relative performance (discrimination) and the absolute performance (calibration) in the validation cohort. An internal validation was conducted as well.

##### Overall performance

The overall performance was assessed with the scaled Brier score. The Brier score can be calculated with Brier = $\frac{1}{N} \sum _{i~=~1}^N(y_i - \hat{y}_i)^2$, with the number of patients *N*, actual outcome *y* and predicted probability $\hat{y}$ for each patient *i* [17]. A perfect prognostic model has a Brier score of 0. Since the Brier score depends on the prevalence of the outcome, the interpretation of the Brier score can be simplified by scaling the Brier score from 0% (non-informative model) to 100% (perfect model): $\text {Brier}_\text {scaled}~=~(1 - \frac{\text {Brier}}{\text {Brier}_\text {max}}) \times 100$, with $\text {Brier}_\text {max}~=~\frac{1}{N} \sum _{i~=~1}^N y_i \times (1 - \frac{1}{N} \sum _{i~=~1}^N y_i)$.

##### Relative performance

The model discrimination is the model’s ability to differentiate between the patients with and without the outcome [17]. Model discrimination was summarized with the concordance *c* statistic and its corresponding 95% confidence interval, calculated with a bootstrap resampling approach. Due to the binary outcome, the *c* statistic is equal to the area under the receiver operating characteristic (ROC) curve (AUC), a summary statistic of the ROC curve. The ROC curve contains the sensitivity on the *y*-axis and 1 - specificity on the *x*-axis. A perfect prognostic model has an AUC of 1; a non-informative prognostic model has an AUC of 0.5.

##### Absolute performance

The agreement between the actual outcome *y* based on a suitable binning and the predicted probability $\hat{y}$ is shown with a calibration plot [17]. A calibration slope smaller than 1 is an indicator for overfitting, or in other words, the predicted probabilities are higher than the observed outcome rates. In R, the function val.prob.ci.2 of the package CalibrationCurves was used to plot the calibration plots and calculate the calibration intercept, calibration slope and AUC. Calibration intercept corresponds to calibration-in-the-large [29].

#### IV) Final prognostic model

The final prognostic model was defined by the model with the best model performance regarding calibration intercept, calibration slope and AUC. The final prognostic model was also fitted to the total cohort (derivation and validation cohort).

#### Software

The statistical analysis was conducted with the statistical software R, version 4.2.0 [30]. A dynamic reporting approach with a fully scripted analysis was chosen. The following R base packages (base, datasets, graphics, grDevices, grid, methods, parallel, splines, stats, stats4, utils) and other packages (abe 3.0.1, abess 0.4.5, bestglm 0.37.3, Cairo 1.5.15, CalibrationCurves 0.1.2, colorspace 2.0.3, doParallel 1.0.17, doRNG 1.8.2, dplyr 1.0.9, foreach 1.5.2, Formula 1.2.4, ggplot2 3.3.6, Hmisc 4.7.0, iterators 1.0.14, lattice 0.20.45, leaps 3.1, libcoin 1.0.9, magrittr 2.0.3, mgcv 1.8.40, missForest 1.5, mvtnorm 1.1.3, nlme 3.1.157, partykit 1.2.15, randomForest 4.7.1.1, readxl 1.4.0, regclass 1.6, rms 6.3.0, rngtools 1.5.2, rpart 4.1.16, skimr 2.1.4, SparseM 1.81, survival 3.3.1, tableone 0.13.2, VGAM 1.1.6, VIM 6.1.1, xtable 1.8.4) were used.

## Results

The characteristics of the patients in this study are given in Table 1. In total, 2799 patients participated in the study. 1588 patients were included in the derivation cohort and 1211 patients in the validation cohort. Fifty-four percent of the patients were male. Missing values occurred rarely, specifically in the candidate predictors body mass index (0.3%), presence of at least one cardiovascular risk factor (0.4%), responsibility for childcare/family members (1.6%), main responsibility for household (0.9%) and perceived stress level at home (0.7%). The following candidate predictors had a SMD between derivation and calibration cohort greater than 0.1: Sex (SMD = 0.105), age at presentation (SMD = 0.122), the severity of acute COVID-19 (SMD = 0.316) and responsibility for childcare/family member (SMD = 0.160). In the derivation cohort, the median age was higher than within the patients of the validation cohort, whereas the IQR was similar. The proportion of women participating was higher than in the validation cohort. In the validation cohort, more patients were admitted to the ward or the ICU/IMC and less patients seemed to be responsible for childcare/family members. The median follow-up time was 162 days (146 to 282) in the derivation cohort and 176 days (127 to 225) in the validation cohort. The REST prevalence was similar between the cohorts, with 21.6% (*n* = 343) in the derivation cohort and 22.1% (*n* = 268) in the validation cohort.

**Table 1:** Study participants characteristics, stratified by data cohort

	Derivation cohort	Geographic validation cohort	SMD	Missing values (%)
*n*	1588	1211		
Female sex (%)	765 (48.2)	520 (42.9)	0.105	0.0
Age at presentation in years (median [IQR])	44.00 [30.00, 56.00]	38.00 [29.00, 56.00]	0.122	0.0
Body mass index in kg/m^2^ (median [IQR])	24.65 [22.27, 28.05]	24.22 [21.77, 27.34]	0.098	0.3
Number of comorbidities (median [IQR])	0.00 [0.00, 1.00]	0.00 [0.00, 1.00]	0.020	0.0
Presence of at least one cardiovascular risk factor (%)	489 (30.9)	361 (29.9)	0.023	0.4
Number of acute COVID-19 symptoms (median [IQR])	5.00 [3.00, 6.00]	5.00 [3.00, 6.00]	0.041	0.0
Severity of acute COVID-19 (%)			0.316	0.0
Outpatients	1395 (87.8)	941 (77.7)		
Ward	147 (9.3)	151 (12.5)		
ICU/IMC	46 (2.9)	119 (9.8)		
Responsibility for childcare/family member (median [IQR])	1.00 [1.00, 4.00]	1.00 [1.00, 3.00]	0.160	1.6
Main responsibility for household (%)			0.026	0.9
No	368 (23.4)	294 (24.5)		
Partners contributed approximately equally	533 (33.9)	398 (33.1)		
Yes or live alone	672 (42.7)	509 (42.4)		
Feeling of stress at home (1 no, 10 max) (median [IQR])	3.00 [2.00, 5.00]	3.00 [2.00, 5.00]	0.084	0.7
Study site				0.0
University Hospital Basel	1588 (100.0)	0 (0.0)		
University Hospital Zurich	0 (0.0)	897 (74.1)		
University Hospital Bern	0 (0.0)	36 (3.0)		
Cantonal Hospital Baden	0 (0.0)	278 (23.0)		

Legend: *ICU/IMC* intensive or intermediate care, *IQR* interquartile range, *SMD* standardized mean difference between derivation and validation cohort

The largest Pearson’s correlation coefficient (*r* = 0.34) was calculated between the candidate predictors age at presentation and number of comorbidities. All Pearson’s correlation coefficients were shown for the derivation cohort in Additional file 1. The highest VIF value was found for age (VIF = 1.48). The final global logistic regression model, including all candidate predictors, was fitted without interactions, because no evidence for an interaction between age and body mass index, age and number of comorbidities, age and responsibility for childcare/family member, sex and presence of at least one cardiovascular risk factor, and body mass index and number of comorbidities was found. Furthermore, there was no evidence for non-linear relationships following graphical display (Additional file 2).

**Fig. 1:**
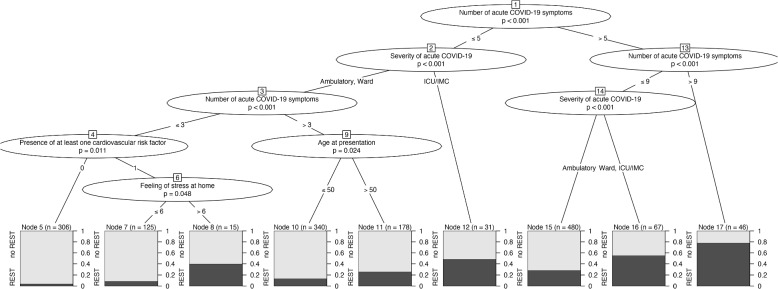
Results of the the model-based recursive partitioning (MBRP) approach

According to ABE and ABESS methods, the same predictors were selected, specifically the number of acute COVID-19 symptoms, severity of the acute COVID-19, feeling of stress at home, age at presentation, sex, presence of at least one cardiovascular risk factor, responsibility for childcare/family member and body mass index. The following five candidate predictors were selected with the MBRP approach (Fig. 1): number of acute COVID-19 symptoms, severity of the acute COVID-19, feeling of stress at home, age and presence of at least one cardiovascular risk factor. The first partitioning variable was the number of acute COVID-19 symptoms. According to the generalized linear model, patients with more than nine symptoms had on average approximately an 80% risk for REST symptoms. Having equal or less than nine symptoms and being an outpatient reduced the risk on average to approximately 28%.

**Fig. 2:**
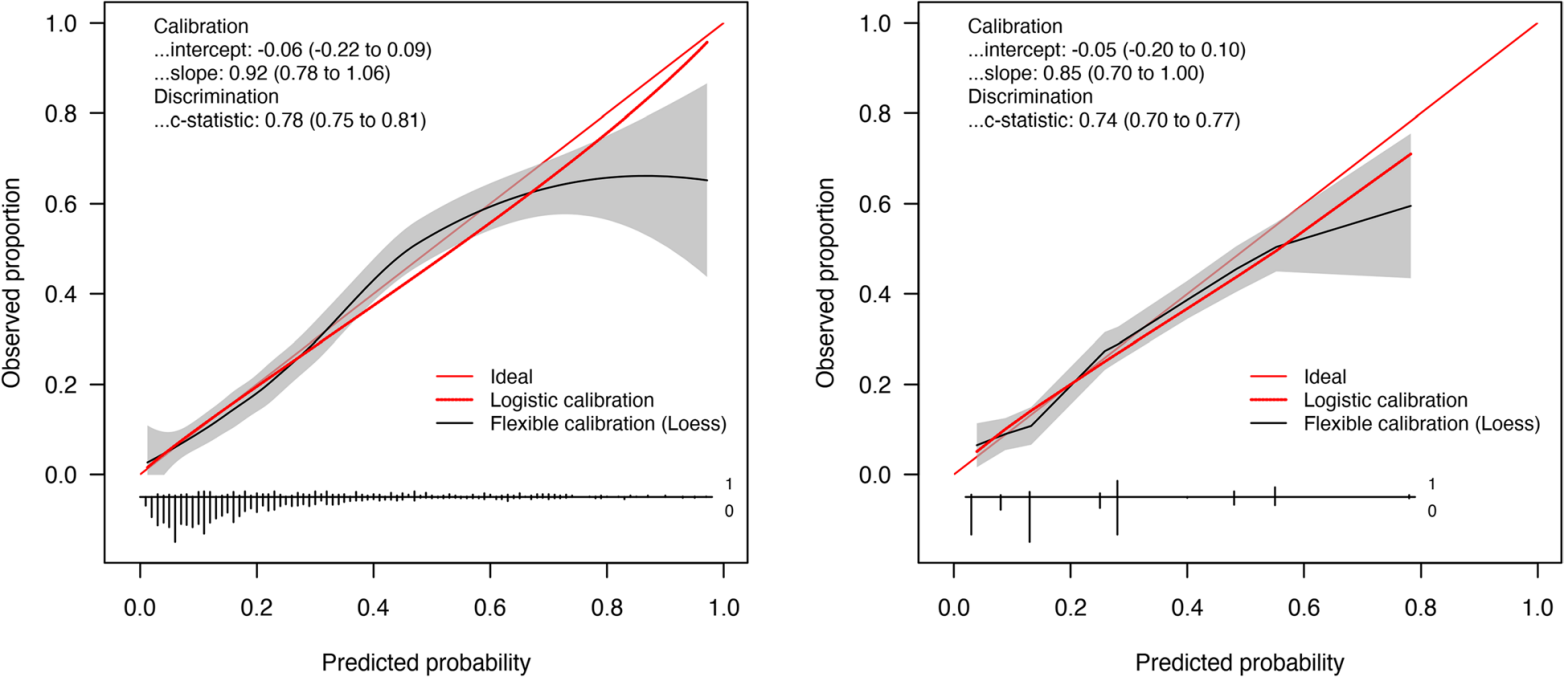
Calibration plots for the logistic regression models $\text {M}_{\text {ABE}}$ = $\text {M}_{\text {ABESS}}$ (left side) and $\text {M}_{\text {MBRP}}$ (right side)

In the following, the results of the validation cohort are given. The Brier score and scaled Brier score of $\text {M}_\text {ABE}$, $\text {M}_\text {ABESS}$ was 0.14 and 18.74%, respectively. In contrast, $\text {M}_\text {MBRP}$ had a Brier score and scaled Brier score of 0.15 and 12.78 %, respectively. The *c* statistic was 0.78 (95% CI: 0.75 to 0.81) for $\text {M}_\text {ABE}$ and $\text {M}_\text {ABESS}$ and 0.74 (95% CI: 0.70 to 0.77) for $\text {M}_\text {MBRP}$. The calibration plots are shown in Fig. 2. The final prognostic model (Table 2) was based on the ABE and ABESS method because model performance (calibration slope and *c*-statistic) was better. The corresponding results of the derivation cohort are given in Additional file 3.

**Table 2:** Logistic regression model based on the ABE and ABESS variable selection, calculated on the derivation cohort

	Estimate	OR	95 % CI for OR
(Intercept)	−4.95		
Number of acute COVID-19 symptoms	0.35	1.42	from 1.33 to 1.51
Severity of acute COVID-19 Ward	0.34	1.40	from 0.90 to 2.20
Severity of acute COVID-19 ICU/IMC	1.74	5.68	from 2.86 to 11.31
Feeling of stress at home	0.13	1.14	from 1.07 to 1.21
Age at presentation [years]	0.01	1.01	from 1.00 to 1.02
Female sex	0.35	1.42	from 1.08 to 1.87
Presence of at least one cardiovascular risk factor	0.35	1.41	from 1.05 to 1.91
Responsibility for childcare/family member	−0.10	0.91	from 0.84 to 0.98
Body mass index [kg/m^2^]	0.02	1.02	from 0.99 to 1.05

Legend: *CI* confidence interval, *OR* odds ratio. Feeling of stress at home ranges from 1 (no stress) to 10 (maximum stress) and responsibility for childcare/family member ranges from 1 (no responsibility/not applicable) to 6 (full responsibility)

The prediction of the REST outcome can be calculated with the following formula:

$$ \begin{aligned} {S}&= {-4.946} \\&\quad {+ 0.349 \times \text {number of acute COVID-19 symptoms}} \\&\quad { + 0.339 \times \text {severity of acute COVID-19 ward}} \\ &\quad { + 1.738 \times \text {severity of acute COVID-19 intensive or intermediate care}} \\ &\quad {+ 0.128 \times \text {feeling of stress at home}} \\ &\quad {+ 0.013 \times \text {age at presentation}} \\ &\quad {+ 0.351 \times \text {female sex}} \\ &\quad {+ 0.346 \times \text {presence of at least one cardiovascular risk factor}} \\ &\quad {- 0.096 \times \text {responsible for childcare/family member}} \\ &\quad {+ 0.022 \times \text {body mass index}}, \end{aligned}$$

with feeling of stress at home ranging from 1 (no stress) to 10 (maximum stress) and responsibility for childcare/family member ranges from 1 (no responsibility/not applicable) to 6 (full responsibility). A patient’s predicted probability $\hat{y}$ for REST symptoms can be calculated with $\hat{y}~=~\frac{exp(S)}{1+exp(S)}$. For comparison purposes, the coefficients were re-estimated on the total cohort (derivation and validation) and the coefficients are given in Additional file 4, resp. Table 3.

In the following, an example for the calculation of the REST outcome is given. The patient had seven acute COVID-19 symptoms and was admitted to the ward during the acute COVID-19 disease. The stress level at home was rated as 4. The patient is 38 years old and a woman, has two cardiovascular risk factors, has no children at home and is not responsible for another family member. The patient has a BMI of 22. The probability for the REST outcome is 50.57% and calculated as follows:

$$ \begin{aligned} {0.023}&= {-4.946} \\&\quad {+ 0.349 \times 7} \\&\quad {+ 0.339 \times \text {1}} \\&\quad {+ 1.738 \times 0} \\&\quad {+ 0.128 \times 4} \\&\quad {+ 0.013 \times 38} \\&\quad {+ 0.351 \times \text {1}} \\&\quad {+ 0.346 \times \text {1}} \\&\quad {- 0.096 \times 0} \\&\quad {+ 0.022 \times 22,} \end{aligned}$$

with $$\frac{exp(0.023)}{1 + exp(0.023)} \times 100 = 50.57$$ %.

## Discussion

In this study, a prognostic model to estimate the probability of common REST symptoms in COVID-19 patients was developed and validated. Long COVID can have serious consequences on daily living, employment, social functioning, mental health and quality of life for months after the acute COVID-19 infection [3]. An early detection of high-risk patients directly after the acute COVID-19 would allow to support patients at an early stage. Interventions such as tailored exercise training, breathing exercise and psychological support could have a positive effect on the further course of the disease and reduce the socio-economic burden of long COVID.

Predictors were selected and estimated based on three methods, namely ABE, ABESS and MBRP. The same predictors were selected with the ABE and ABESS method. The model performance (in regard to *c* statistic and calibration slope) of $\text {M}_{\text {ABE}}$ = $\text {M}_{\text {ABESS}}$ was better compared to $\text {M}_{\text {MBRP}}$. The calibration slope for $\text {M}_{\text {ABESS}}$ = $\text {M}_{\text {ABE}}$ indicated slight overfitting. In particular, higher probabilities were overestimated. The MBRP approach allows physicians to determine a patient’s risk for long COVID by following a decision tree and may for this reason be easier to implement in clinical practice. The decision tree contained nine subtrees for predicting the REST outcome. Patients within a subtree had the same risk estimate for REST symptoms, resulting in nine different predicted probabilities.

To the best of the authors’ knowledge, two prognostic models for the prognosis of long COVID were published so far [31, 32]. In the first published prognostic model [31], the probability for long COVID lasting for at least 28 days was calculated with the predictor number of acute COVID-19 symptoms, age and sex based on self-reported data of 2149 patients from Sweden, the UK and the USA. The second published prognostic model [32] included age, number of acute COVID-19 symptoms, history of asthma bronchiale, the antibodies total Immunoglobulin (Ig) M, IgG3 and the interaction between IgM and IgG3. The model was developed based on 134 patients (85 patients with the long COVID outcome) from four Swiss hospitals in and around Zurich. Model validation was performed with data of 389 patients (212 patients with long COVID). In the derivation cohort, 53.9% patients with a mild COVID-19 course and 82.2% patients with a severe COVID-19 course suffered from long COVID. In the validation cohort, 54.7% patients reported long COVID. Long COVID was defined similarly in both publications. The prognostic models for the prognosis of long COVID mentioned above could not be validated with our data due to different long COVID symptoms and follow-up time and because immunoglobulins such as IgM and IgG3 were not assessed in our cohort.

Our study has several strengths. These include the large number of patients and relevant candidate predictors. Additionally, the geographic validation can be considered as an external validation of the model. Further, the percentage of missing data was low. The limitations of the study were the following: patients with a wide variety of disease severity (from outpatient to ICU/IMC) were included. Furthermore, there was heterogeneity in the follow-up time, which might have reduced the performance of the prognostic model. However, the median follow-up time was similar in derivation and validation cohort. Nevertheless, theoretically, it is possible that some patients have suffered from persistent REST symptoms but have since recovered from their illness. At the design phase of this study, however, no clear guidelines regarding timeframes in the context of long COVID existed. For this reason, the minimum follow-up period was 60 days (corresponding to 8.6 weeks) in our study. As a sensitivity analysis, the recent timeframe definition of post-COVID, i.e. persisting symptoms lasting longer than 12 weeks, was applied post-hoc, leading to an exclusion of 54 patients from the analysis. The results were comparable as the same variables were selected and the coefficients of the logistic regression model were similar.

Our study has implication for future research in the sense that the prognostic model developed and validated in this study should be validated in new patients (e.g. from different settings, different COVID-19 mutations, and other countries). Furthermore, different presentation methods of the model could be developed. Before implementing the developed and validated prognostic model for estimating the risk of REST symptoms in daily clinical practice, it is advisable to evaluate the model impact. This might be done with a randomized ‘impact study’ [33]. The aim of the impact study would be to investigate if the application of the prognostic model is leading to better patient outcomes by earlier detection or reduction of REST symptoms.

## Conclusions

The proposed model was validated to identify COVID-19-infected patients at high risk for REST symptoms. Before implementing the prognostic model in daily clinical practice, the conduction of an impact study is recommended.

## Supplementary information

**Additional file 1.** Histogram, smooth function and Pearson's correlation coefficients for continuous candidate predictors.

**Additional file 2.** Partial plots for continuous candidate predictors of the global logistic generalized additive model. The *y*-axis contains the REST outcome on the log-odds scale $(ln(\frac{\hat{y}}{1-\hat{y}}))$, with the patient's predicted probability $\hat{y}$  of developing REST symptoms.

**Additional file 3.** Calibration plots for the logistic regression models M_ABE_ = M_ABESS_ (left side) and M_MBRP_ (right side) based on the derivation cohort. The Brier score and scaled Brier score of M_ABE_, M_ABESS_ was 0.14 and 17.01%, respectively. In contrast, M_MBRP_ had a Brier score and scaled Brier score of 0.14 and 15.41%, respectively.

**Additional file 4.** Logistic regression model based on the ABE and ABESS variable selection, calculated on the total cohort.

## Data Availability

In case of non-commercial reasons and upon reasonable requests, anonymised data will become available after signing a data access agreement. Requests can be sent to Prof. Catherine Gebhard, MD, PhD, Center for Molecular Cardiology, University of Zurich and Department of Nuclear Medicine, University Hospital Zurich, Rämistrasse 100, 8091 Zurich, Switzerland, catherine.gebhard@usz.ch. Statistical programming code will be made available upon request.

## Abbreviations

ABE: Augmented backward elimination
ABESS: Adaptive best-subset selection
AIC: Akaike information criterion
AUC: Area under the curve
BASEC: Business Administration System for Ethics Committees
BIC: Bayesian information criterion
COVID-19: Coronavirus disease 2019
ICU: Intensive care unit
Ig: Immunoglobulin
IgG3: Immunoglobulin G3
IgM: Immunoglobulin M
IMC: Intermediate care
IQR: Interquartile range
MBRP: Model-based recursive partitioning
PCR: Polymerase chain reaction
REST: Reduced exercise tolerance/reduced resilience, shortness of breath and/or tiredness
ROC: Receiver operating characteristic
SARS-CoV-2: Severe acute respiratory syndrome coronavirus 2
SMD: Standardized mean difference
TRIPOD: Transparent Reporting of a Multivariable Prediction Model for Individual Prognosis or Diagnosis
VIF: Variance inflation factor
WHO: World Health Organization
