# Supplementary figures and images for: Development and validation of a prognostic model for the early identification of COVID-19 patients at risk of developing common long COVID symptoms
Source: Diagn Progn Res. 2022 Nov 17;6:22. doi: 10.1186/s41512-022-00135-9 (PMC9668400; doi:10.1186/s41512-022-00135-9)

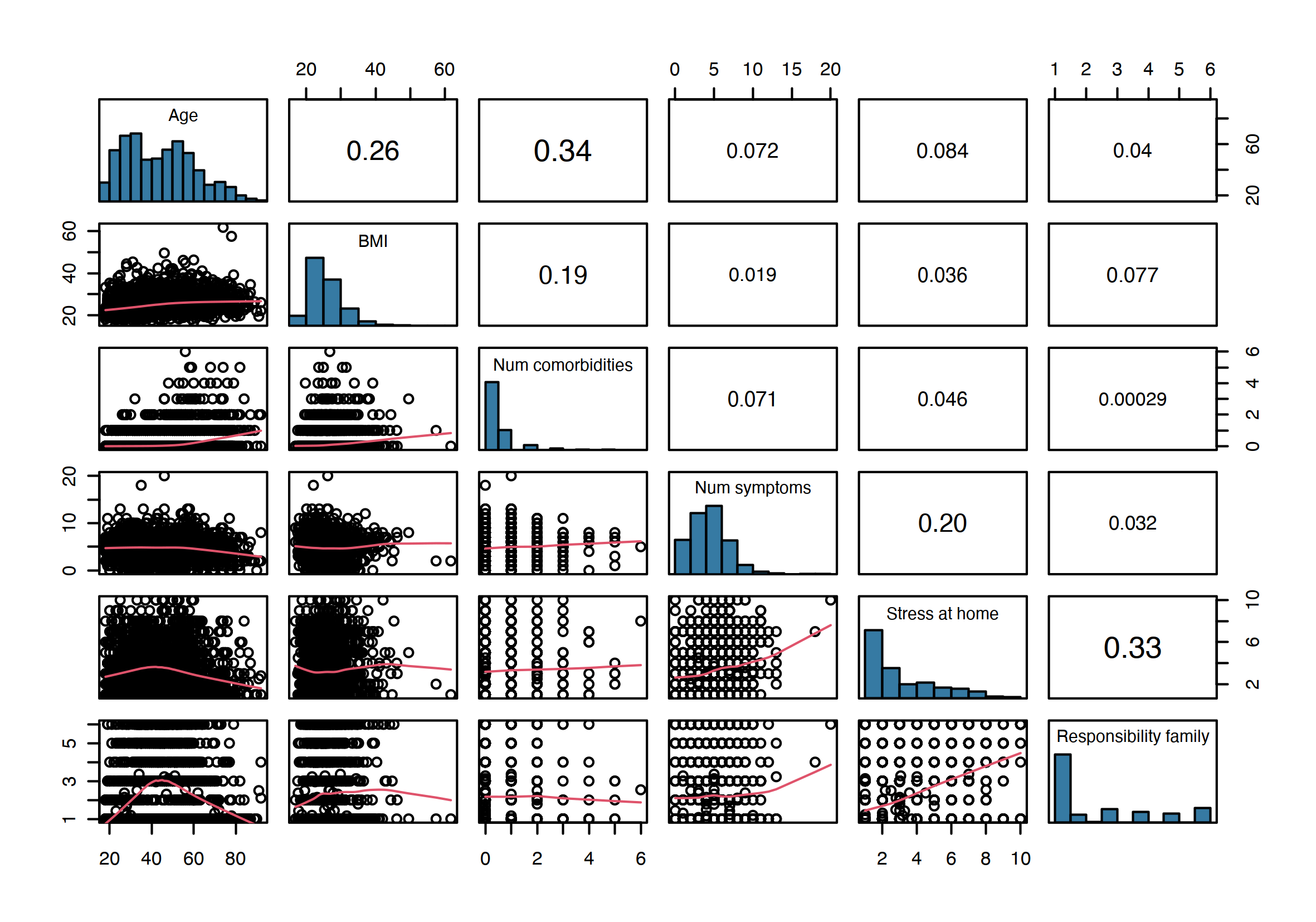

Supplement: Supplementary file 1 — Additional file 1. Histogram, smooth function and Pearson's correlation coefficients for continuous candidate predictors. [file 41512_2022_135_MOESM1_ESM.png]

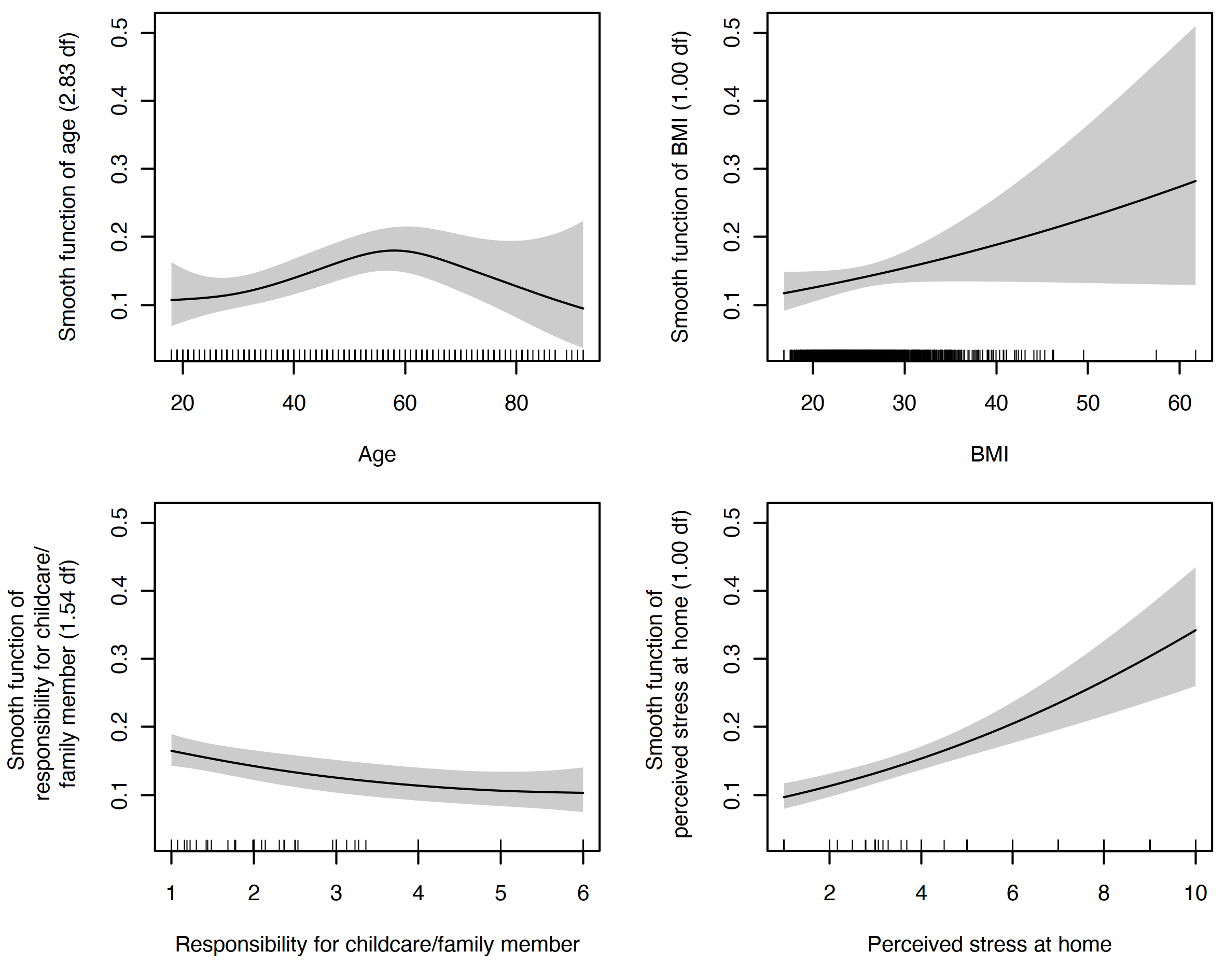

Supplement: Supplementary file 2 — Additional file 2. Partial plots for continuous candidate predictors of the global logistic generalized additive model. The y-axis contains the REST outcome on the log-odds scale $(ln(\frac{\hat{y}}{1-\hat{y}}))$(ln(y^1-y^)), with the patient's predicted probability $\hat{y}$y^ of developing REST symptoms. [file 41512_2022_135_MOESM2_ESM.png]

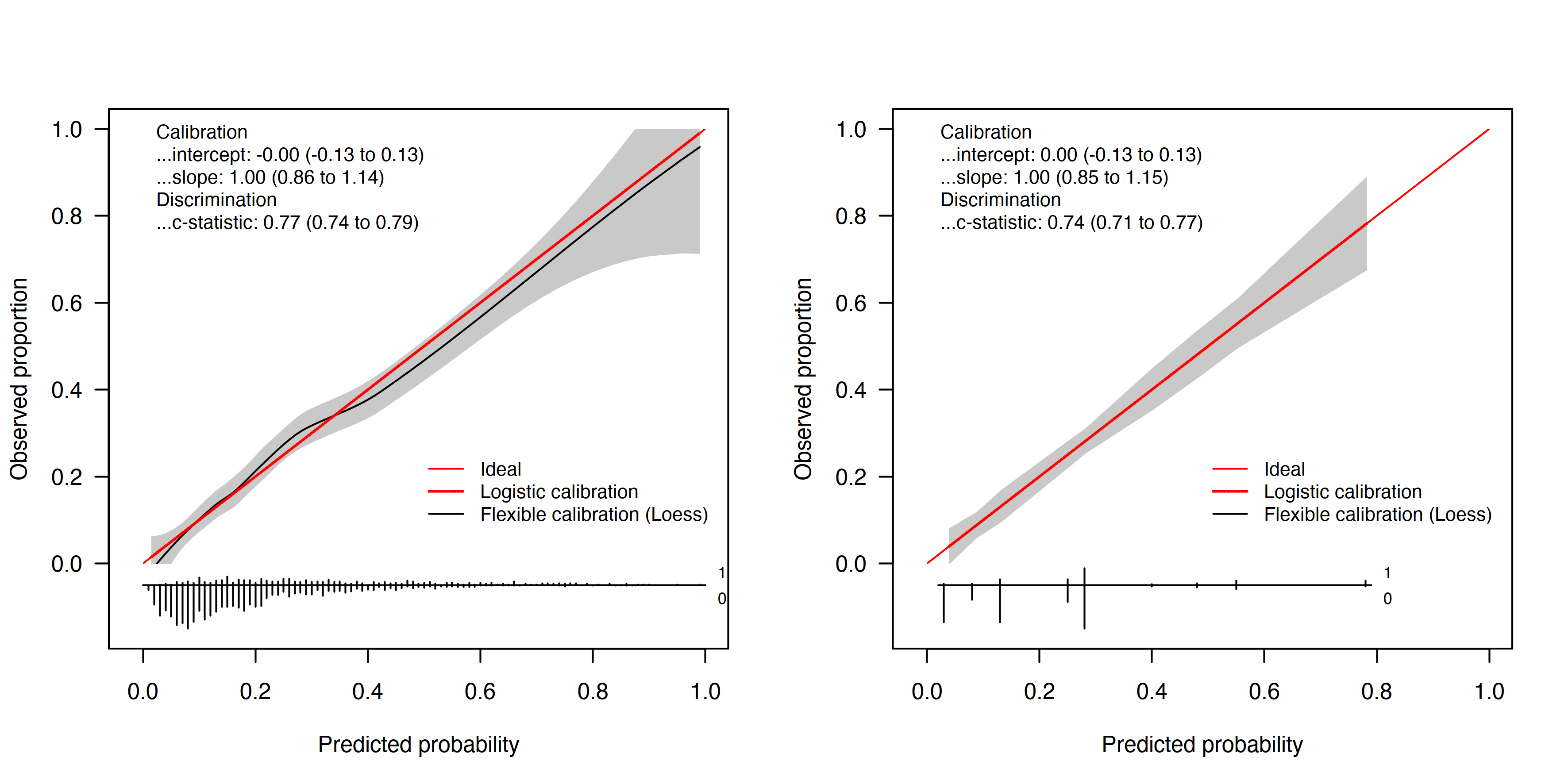

Supplement: Supplementary file 3 — Additional file 3. Calibration plots for the logistic regression models MABE = MABESS (left side) and MMBRP (right side) based on the derivation cohort. The Brier score and scaled Brier score of MABE, MABESS was 0.14 and 17.01%, respectively. In contrast, MMBRP had a Brier score and scaled Brier score of 0.14 and 15.41%, respectively. [file 41512_2022_135_MOESM3_ESM.png]
