# Supplementary material for: Development and validation of a prognostic model for the early identification of COVID-19 patients at risk of developing common long COVID symptoms
Source: Diagn Progn Res. 2022 Nov 17;6:22. doi: 10.1186/s41512-022-00135-9 (PMC9668400; doi:10.1186/s41512-022-00135-9)
Supplement: Supplementary file 4 — Additional file 4. Logistic regression model based on the ABE and ABESS variable selection, calculated on the total cohort. [file 41512_2022_135_MOESM4_ESM.pdf]

Table 3 Logistic regression model based on the ABE and ABESS variable selection, calculated on the total cohort.

	Estimate	OR	95 % CI for OR
(Intercept)	-4.98		
Number of acute COVID-19 symptoms	0.34	1.40	from 1.34 to 1.47
Severity of acute COVID-19 Ward	0.46	1.58	from 1.15 to 2.17
Severity of acute COVID-19 ICU/IMC	1.38	3.97	from 2.69 to 5.84
Feeling of stress at home	0.11	1.12	from 1.07 to 1.18
Age at presentation [years]	0.01	1.01	from 1.00 to 1.02
Female sex	0.35	1.42	from 1.15 to 1.75
Presence of at least one cardiovascular risk factor	0.45	1.58	from 1.25 to 1.98
Responsibility for childcare/family member	-0.11	0.89	from 0.84 to 0.95
Body mass index [kg/m^2]	0.03	1.03	from 1.01 to 1.05

Legend: Confidence interval (CI); odds ratio (OR). Feeling of stress at home ranges from 1 (no stress) to 10 (maximum stress) and responsibility for childcare/family member ranges from 1 (no responsibility/not applicable) to 6 (full responsibility).
